# OD26 Comparing Health Technology Developers’ Proposed Indication To An Estimated Indication Generated By An International Horizon Scanning Database

**DOI:** 10.1017/S0266462324001570

**Published:** 2025-01-07

**Authors:** Marie Harte, Caitríona Ní Choitir, Heather Eames, Roisin Adams, Laura McCullagh, Lesley Tilson, Daisy Duell, Irina Odnoletkova, Anna Bergkvist Christensen, Marie Persson, Helle Bräuner, Marcus Guardian, Alzbeta Alzbeta Tuckova, Brian Wilkinson, Beth Kuzmak, John Shaw, Craig Boyce, Prashanth Palakollu, Eileen Erinoff

## Abstract

**Introduction:**

Detail on a technology’s projected therapeutic use is required for horizon scanning. The International Horizon Scanning Initiative (IHSI) database will utilize natural language processing (NLP) augmented by human curation to generate an estimated indication for technologies in development. We compared the estimated indication, generated as a test-set for NLP, with health technology developers’ (HTDs) proposed indications identified from Ireland’s horizon scanning system (HSS).

**Methods:**

Eight oncology technologies common to both Ireland’s HSS and the IHSI database were analyzed. The analysis included unlicensed technologies in late-stage development that have not submitted a European marketing authorization application. Ireland’s HSS receives data on proposed indications for technologies from HTDs. IHSI database curators extract and convert terms from clinical trials into structured inputs (condition, combination therapy, stage of disease, place in treatment, patient/disease-specific subgroups) to produce an estimated indication for a technology. We sought to identify, by structured input, the degree of alignment between HTDs’ proposed indications with the IHSI database’s estimated indication.

**Results:**

There was 100 percent alignment between the HTD’s proposed indication and the estimated indication generated in the IHSI database for five of the eight included technology records. There was 83 percent alignment for two records and 67 percent alignment for one record. Across all records there was full alignment on condition, combination therapy details, patient-specific subgroup, disease-specific subgroup, and place in treatment. Stage of disease was the only element where data was either not generated for the IHSI database’s estimated indication, not aligned with the HTD’s proposed indication, or reported in an incorrect field.

**Conclusions:**

There is a high degree of alignment between an HTD-proposed indication and the IHSI-estimated indication. The processes for generating an estimated indication will involve both NLP-generation and human co-curation. The current (curator-selected) elements are being used to train the NLP engine. Thereafter, the engine will process clinical trial data to surface tags for human selection to generate the structured inputs.

